# Supplementary material for: Progression-Free and Overall Survival of First-Line Treatments for Advanced Renal Cell Carcinoma: Indirect Comparison of Six Combination Regimens
Source: Cancers (Basel). 2023 Mar 29;15(7):2029. doi: 10.3390/cancers15072029 (PMC10093553; doi:10.3390/cancers15072029)
Supplement: Supplementary file 1 [file cancers-15-02029-s001.zip › cancers-2256486-supplementary.pdf]

cancers MDPI

## Supplementary Materials

**This Supplementary Materials includes the following material:**

- **Table S1.** Comparison of PFS and OS between combination treatments and sunitinib.

- **Figure S1.** Forest plots of HRs with 95%CI for PFS and OS of the six trials against pooled sunitinib controls

- **Figure S2:** Visual representation of indirect treatment comparisons for PFS.

- **Figure S3.** Visual representation of indirect treatment comparisons for OS.

**Table S1.** Results of statistical analysis of the comparison between combination treatments and sunitinib. Upper panel: PFS, Lower panel: OS. Each combination treatment has been compared with the pooled group of 6 sunitinib control arms.

| **Combination treatment** | **Median PFS in months (95% CI)** | | **HR (95% CI) §** | **P** |
|---|---|---|---|---|
| | **combination treatment** | **sunitinib*** | | |
| atezolizumab+ bevacizumab [11] | 11.45 (9.87 to 13.5) | 9.39 (8.12 to 10.10) | 0.9822 (0.8637 to 1.117) | 0.784 |
| avelumab+ axitinib [12] | 13.85 (10.99 to 16.7) | 8.17 (6.81 to 10.01) | 0.8029 (0.7012 to 0.9193) | 0.00148 |
| nivolumab+ ipilimumab [13] | 14.29 (12.02 to 18.8) | 14.01 (11.80 to 16.34) | 0.6718 (0.596 to 0.7571) | <0.001 |
| lenvatinib+ pembrolizumab [14] | 24.52 (22.02 to 29.5) | 9.83 (7.35 to 10.85) | 0.4959 (0.4219 to 0.583) | <0.001 |
| nivolumab+ cabozantinib [15] | 17.12 (13.01 to 20.2) | 8.67 (7.16 to 9.99) | 0.6943 (0.5988 to 0.805) | <0.001 |
| pembrolizumab + axitinib [16] | 16.99 (14.21 to 21.1) | 11.49 (10.10 12.75) | 0.701 (0.6142 to 0.8001) | <0.001 |

*The median of PFS in the pooled group of 6 sunitinib control arms is 9.92 months (9.53 to 10.09).
§ Each combination treatment has been compared with the pooled group of 6 sunitinib control arms.

| **Combination treatment** | **Median OS in months (95% CI)** | | **HR (95% CI) §** | **P** |
|---|---|---|---|---|
| | **combination treatment** | **sunitinib*** | | |
| atezolizumab+ bevacizumab [11] | 35.5 (28.9 to NA) | 35.1 (27.7 to NA) | 1.0818 (0.9148 to 1.2794) | 0.358 |
| avelumab+ axitinib [12] | NA (30.0 to NA) | NA (27.9 to NA) | 0.8562 (0.6984 to 1.0496) | 0.135 |
| nivolumab+ ipilimumab [13] | 59.2 (48.0 to 70.3) | 41.2 (35.6 to 49.6) | 0.7338 (0.6348 to 0.8482) | <0.001 |
| lenvatinib+ pembrolizumab [14] | NA (NA to NA) | NA (NA to NA) | 0.5731 (0.4522 to 0.7263) | <0.001 |
| nivolumab+ cabozantinib [15] | 37.9 (36.3 to NA) | 34.3 (29.6 to NA) | 0.8516 (0.6946 to 1.0440) | 0.122 |
| pembrolizumab + axitinib [16] | NA (NA to NA) | 36.9 (33.4 to NA) | 0.6601 (0.5369 to 0.8116) | <0.001 |

**Figure S1.** Forest plots of HRs with 95%CI for PFS and OS of the six trials against pooled sunitinib controls

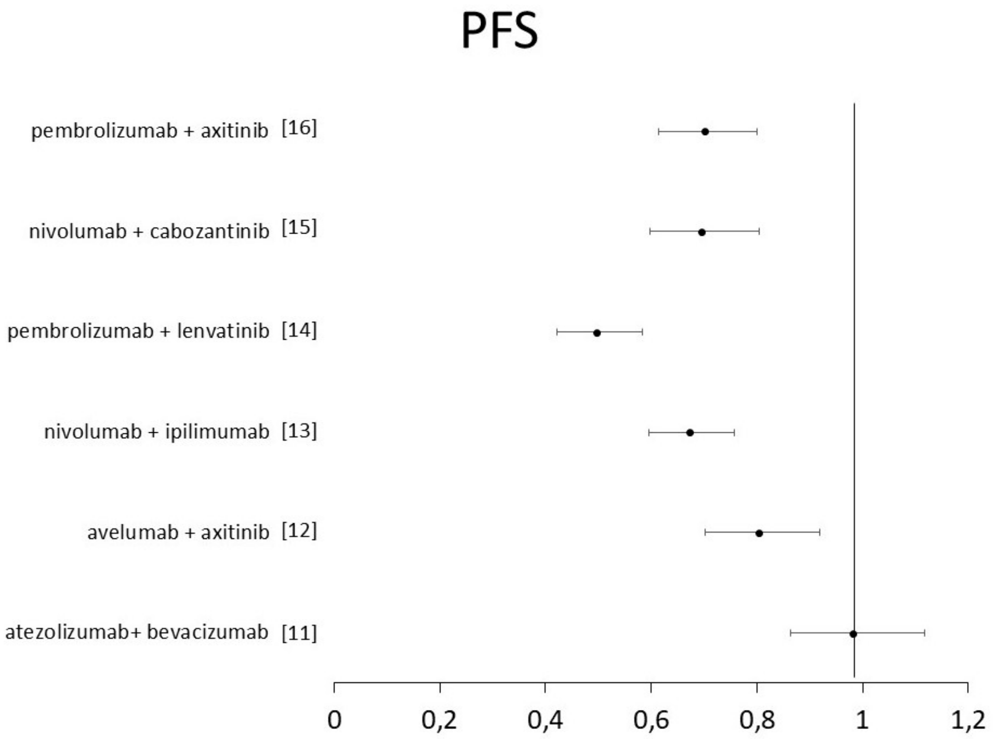


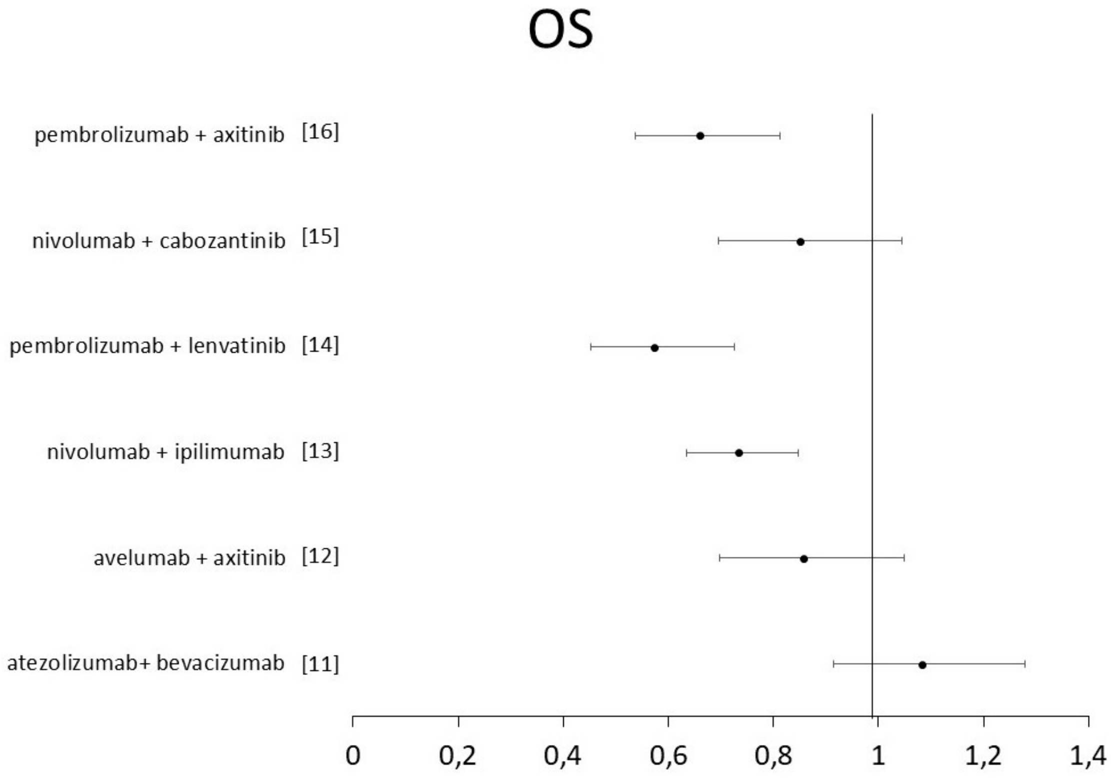

**Figure S2.** Visual representation of indirect treatment comparisons for PFS. All head-to-head comparisons are represented in form A vs. B, where treatment A is in the first column on the left and treatment B is in the second column, and so on. Green: A is superior to B; Red: A is inferior to B; Light Blue: no significant difference between A and B.

| ↓ vs. → | atezolizumab+ bevacizumab | avelumab+ axitinib | nivolumab+ ipilimumab | pembrolizumab + lenvatinib | nivolumab+ cabozantinib | pembrolizumab + axitinib |
|---|---|---|---|---|---|---|
| atezolizumab+ bevacizumab | | | | | | |
| avelumab+ axitinib | | | | | | |
| nivolumab+ ipilimumab | | | | | | |
| Pembrolizumab + lenvatinib | | | | | | |
| nivolumab+ cabozantinib | | | | | | |
| pembrolizumab + axitinib | | | | | | |

**Figure S3.** Visual representation of indirect treatment comparisons for OS. All head-to-head comparisons are represented in the form of A vs. B, where treatment A is in the first column on the left and treatment B is in the second column, and so on. Green: A is superior to B; Red: A is inferior to B; Light Blue: no significant difference between A and B.

| ↓ vs. → | atezolizumab+ bevacizumab | avelumab+ axitinib | nivolumab+ ipilimumab | pembrolizumab + lenvatinib | nivolumab+ cabozantinib | pembrolizumab + axitinib |
|---|---|---|---|---|---|---|
| atezolizumab+ bevacizumab | | | | | | |
| avelumab+ axitinib | | | | | | |
| nivolumab+ ipilimumab | | | | | | |
| pembrolizumab + lenvatinib | | | | | | |
| nivolumab+ cabozantinib | | | | | | |
| pembrolizumab + axitinib | | | | | | |
